# Factors influencing health care utilisation among Aboriginal cardiac patients in central Australia: a qualitative study

**DOI:** 10.1186/1472-6963-13-83

**Published:** 2013-03-06

**Authors:** Stella Artuso, Margaret Cargo, Alex Brown, Mark Daniel

**Affiliations:** 1 Social Epidemiology and Evaluation Research Group, School of Population Health, University of South Australia, Adelaide, Australia; 2 Baker IDI Heart and Diabetes Institute, Alice Springs, Australia; 3 Department of Medicine, St Vincent’s Hospital, The University of Melbourne, Melbourne, Australia

**Keywords:** Health care utilisation, Facilitators/barriers to care, Cardiovascular disease, Indigenous, Aboriginal

## Abstract

**Background:**

Aboriginal Australians suffer from poorer overall health compared to the general Australian population, particularly in terms of cardiovascular disease and prognosis following a cardiac event. Despite such disparities, Aboriginal Australians utilise health care services at much lower rates than the general population. Improving health care utilisation (HCU) among Aboriginal cardiac patients requires a better understanding of the factors that constrain or facilitate use. The study aimed to identify ecological factors influencing health care utilisation (HCU) for Aboriginal cardiac patients, from the time of their cardiac event to 6–12 months post-event, in central Australia.

**Methods:**

This qualitative descriptive study was guided by an ecological framework*.* A culturally-sensitive illness narrative focusing on Aboriginal cardiac patients’ “typical” journey guided focus groups and semi-structured interviews with Aboriginal cardiac patients, non-cardiac community members, health care providers and community researchers. Analysis utilised a thematic conceptual matrix and mixed coding method. Themes were categorised into *Predisposing, Enabling, Need* and *Reinforcing* factors and identified at *Individual, Interpersonal, Primary Care* and *Hospital System* levels.

**Results:**

Compelling barriers to HCU identified at the *Primary Care* and *Hospital System* levels included communication, organisation and racism. *Individual* level factors related to HCU included language, knowledge of illness, perceived need and past experiences. Given these individual and health system barriers patients were reliant on utilising alternate family-level supports at the *Interpersonal* level to enable their journey.

**Conclusion:**

Aboriginal cardiac patients face significant barriers to HCU, resulting in sub-optimal quality of care, placing them at risk for subsequent cardiovascular events and negative health outcomes. To facilitate HCU amongst Aboriginal people, strategies must be implemented to improve communication on all levels and reduce systemic barriers operating within the health system.

## Background

Aboriginal Australians suffer from poorer overall health compared to the general Australian population, particularly in terms of cardiovascular disease (CVD) and prognosis following a cardiac event. CVD is the primary cause of early adult mortality among Aboriginal Australians [1] and the single greatest contributor to morbidity in the Northern Territory [2]. Premature mortality resulting from CVD is a major contributor to the greater than 10 year life expectancy gap existing between Aboriginal and non-Aboriginal Australians [3].

Few cardiac services and specialists are available in rural and remote areas of Australia where many Aboriginal people live, and accessibility is limited for those wanting such services [4]. But despite such disparities in CVD morbidity and mortality, Aboriginal people utilise health services at lower rates than non-Aboriginal people [4,5]. In the Northern Territory, Aboriginal patients have higher cancellation/non-attendance rates for scheduled appointments and self-discharge against medical advice [6]. It has been reported that Aboriginal people utilising health services experience a wide range of barriers including communication problems, institutional racism, lack of cultural awareness and loneliness [7-11]. Other issues, such as competing priorities, lack of Aboriginal health workers and continuity of health services have been further identified by Aboriginal people as barriers to participation in cardiac rehabilitation programs [12]. A recent review of cardiac rehabilitation programs highlights their lack of cultural appropriateness [13].

In central Australia, where limited services are available and need is high, just a few studies have addressed the issues affecting HCU amongst Aboriginal cardiac patients [14-16]. Maloney (2005) explored the impact of previous experiences on Aboriginal and non-Aboriginal patients’ time to seek tertiary care after a cardiac event. Although Aboriginal patients were as likely to seek tertiary care as were non-Aboriginal patients, intercultural communication problems and fear delayed HCU. An exploratory study found that in remote areas fewer Aboriginal relative to non-Aboriginal cardiac clients travelled to tertiary referral centres for specialist care (personal communication, Brown A, November 2006) despite such services being known to improve survival [17]. An action research project illustrated how small and inexpensive improvements in cultural understanding and communication can increase rural Aboriginal patients’ decisions to seek cardiac care in metropolitan hospitals [15]. Studies exploring Aboriginal cardiac patients’ journey through the healthcare system at multiple levels, and with multiple perspectives, to our knowledge, have not yet been published.

This study aimed to identify the ecological factors influencing HCU amongst Aboriginal cardiac patients from the perspectives of: a) Aboriginal cardiac patients, b) non-cardiac community members who supported Aboriginal family members with CVD, and c) health care providers and community researchers. In order to address HCU patterns among Aboriginal cardiac patients, we asked “*What are the factors influencing HCU following a cardiovascular event among Aboriginal people in central Australia?”* Multiple perspectives were sought to discern a broad range of barriers and facilitators to HCU and to enable a comprehensive understanding of factors influencing Aboriginal cardiac patients in achieving cardiac care.

### Research design and ecological conceptual framework

A fundamental qualitative descriptive design [18] was used to guide the study. This study design provides a rich and straight description of an experience or event with researchers staying closer to the surface of their data in interpreting the meaning of experiences and events. A qualitative descriptive design is appropriate for providing pragmatic answers to questions of interest to practitioners and policy-makers. An *a priori* ecological conceptual framework was developed based on pre-existing models used to explain the factors related to HCU.

Population characteristics, *predisposing, enabling,* and *need* described by Andersen [19] and *reinforcing factors* proposed by Green & Kreuter [20] were combined and resulted in four general conceptual themes presumed to influence HCU through impacts on individual, collective and organisational behaviours.

For the purposes of this study, *predisposing factors* were defined as any factor explaining an individual’s decision to use health services based on their preferences or related experiences. *Enabling factors* were elements or situations that influenced utilisation behaviour. *Reinforcing factors* acted as a “reward”, incentive or disincentive following HCU to encourage or discourage the continuation of this behaviour. Finally, *need* reflected an individual’s perceived need to use heath care services based on their perceived illness and/or clinician’s evaluation of their illness. These factors were examined in relation to *Individual* patient characteristics, patients’ *Interpersonal* support*, Primary Care* and *Hospital* level systems [21]. The *Individual* level referred to patient level characteristics, such as language skills or health beliefs and attitudes. The *Interpersonal* level referred to the support system surrounding individuals, which may have influenced HCU, such as family support. Factors at the *Primary Care* and *Hospital System* levels referred to elements influencing utilisation in regards to the health system itself, such as staff’s cultural awareness. HCU is viewed as a function of these factors, influencing an individual’s decision and ability to use health services. HCU in turn impacts on individuals’ health outcomes. This framework acknowledges that while individuals make their own choices regarding HCU, they are often influenced by contextual factors that go beyond personal choice. To understand HCU, models need to acknowledge and incorporate contextual or system’s level factors.

## Methods

### Study setting

The project was based in Alice Springs, the traditional country of the Arrernte people. Aboriginal people represent 17% of the estimated 28,000 persons living in this town. Study participants were recruited from the town of Alice Springs, six town camps located along the fringes of town, as well as two remote area communities located within a 200 km distance from town.

In town, primary health services include both privately- and publicly-funded services, with Aboriginal clients principally using the latter. The sole regional hospital has no coronary care or on-site cardiology specialist. There is limited in-hospital and post-discharge rehabilitation services provided by an independent NGO. Patients requiring cardiac investigation are routinely transferred, some 1,500 km to tertiary hospitals in Adelaide, South Australia.

Although most remote area communities have access to on-site primary care clinics, town camps do not have on site medical services. Health specialists visit remote area communities annually or biannually, but no cardiac rehabilitation is available.

### Data collection

Participants were recruited from September to December 2006 and comprised of cardiac patients (*n* = 7), non-cardiac community members (*n* = 15), and health care providers and community researchers (*n* = 12). Participants were purposively sampled based on knowledge and experiences with the health system, and willingness to share their stories [22]. Table 1 summarises participant characteristics.

**Table 1:** Participant characteristics

**Participants**	**Eligibility criteria**^**a**^	**Data collection type**	***n***	**Gender**	**Origin**	**Age range**	**Residence**
Aboriginal cardiac patients	Cardiac event 12/2005 -06/2006	Semi- structured Interviews	7	3 M	7 I	38–65	1 T
				4 F			4 TC
							2 RAC
Health care providers & community researchers	Experience in Aboriginal health & research	Unstructured interviews	6	4 M	2 I	30–56	5 TC
				2 F	4 NI		1 RAC
		Focus groups	6	3 M	3 I	30–49	4 T
				3 F	3 NI		1 TC
							1 RAC
Non-cardiac community members	Experience supporting family members with CVD	Focus group- F1	6	6 F	6 I	24–66	3 T
							3TC
		Focus group- F2	4	4 F	4 I	28–66	4TC
		Focus group- M	3	3 M	3 I	30–65	3TC
							1RAC
		Semi-structured interview	2	1 M	2 I	40–54	1 T
				1 F			1TC
**Total**			34	13 M	27 I		16 T
				21 F	7 NI		13 TC
							5 RAC

*Notes*: ^a^ All participants must be18 years or older and living in, or within, 200 km of Alice Springs.

M = male; F = female; I = Indigenous; NI = Non-Indigenous; T = town; TC = town camp; RAC = remote area community; CVD = cardiovascular disease.

Data collection occurred in two phases and under the guidance of a cultural mentor and field supervisor (AB). In Phase One, unstructured interviews, lasting between 45–60 minutes, were carried out with health care providers and community researchers to develop a culturally sensitive illness narrative (*n* = 6). Participant responses were noted manually. The illness narrative recreated an Aboriginal cardiac patient’s “typical” journey in their utilisation of health services from the time of a cardiac event to 6–12 months post-event. The intent was for the narrative to make it easier for participants to share their stories during interviews. Story telling is aligned with the strong oral tradition of Aboriginal cultures [23].

Phase Two was comprised of focus groups and semi-structured interviews. Four focus groups were conducted; three gender-specific groups with non-cardiac community members ranging from three to six participants and one combined group of health care providers and community researchers (*n* = 6). Focus groups were facilitated by an Aboriginal community member, and an Aboriginal and non-Aboriginal author. Nine semi-structured interviews were conducted with Aboriginal cardiac patients (*n* = 7) and non-cardiac community members (*n* = 2). A local non-Aboriginal community researcher (well-accepted amongst community members) and the first author conducted interviews at participant’s homes. Family members assisted with communication in English when required. Focus groups and semi-structured interviews were conducted in English and lasted between 60–120 and 45–60 minutes, respectively. Sessions were audio taped and transcribed.

This study was approved by the Central Australian Human Research Ethics Committee in Alice Springs and the Comites d’evaluation scientific et d’éthique de la recherché, Faculté de medecine, Université de Montréal, Canada.

### Data analysis

Qualitative data analysis utilised Miles and Huberman’s thematic conceptual matrix (TCM) [24] and a mixed coding method combining deductively and inductively derived codes [25]. Analysis was guided by the deductive codes in the ecological conceptual framework. Codes in the columns of the TCM represented the *Individual*, *Interpersonal* Support System, *Primary Care System* and *Hospital System*. Codes in the rows of the TCM represented 4 conceptual themes: *Predisposing*, *Enabling*, *Reinforcing* factors and *Need*.

Prior to analysis, each transcript was read twice and a summary was prepared to identify main ideas [26,27]. Data analysis started by coding two focus groups with Aboriginal women. Text segments (or meaningful units) in the transcripts were compared and contrasted, assigned inductive codes and listed in the TCM. For example, the inductive code “mistrust” appears in the matrix under the deductive codes of *“individual”* and *“predisposing”.* Coding continued until all transcripts were analysed. Data were managed using Atlas-ti software [28]. Coded data were then verified through counter-coding, a process where the analyst refers back to the transcripts and “blindly” codes with the aid of the lexicon (a precise document explaining the coding rules and definitions). Six of the 13 primary documents were randomly selected and counter-coded resulting in an intra-rater reliability of 84% (percentage agreement) [25]. Counter-coding enabled re-definition of the codes where problems of conceptual validity occurred and allowed for necessary adjustments. Cross-cultural verification and opportunistic member checking [26] were applied with consenting participants. Conceptual meanings behind some words were verified to counteract potential misinterpretations and augment cross-cultural understanding.

## Results

**Figure 1:**
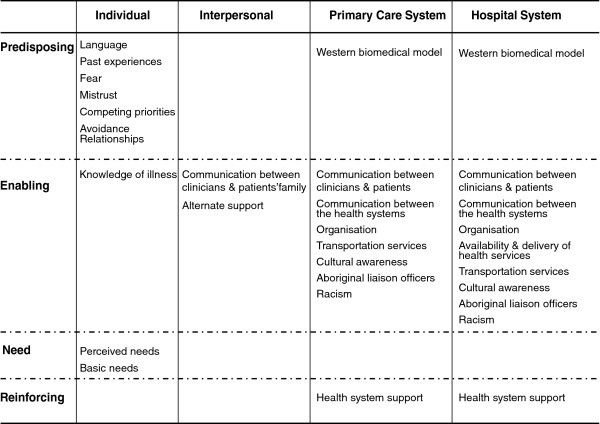
Factors influencing HCU among Aboriginal cardiac patients in central Australia.

Stories from cardiac patients, non-cardiac community members, health care providers and community researchers offered unique, converging or complementary perspectives which informed the development of a culturally sensitive matrix that articulates the factors influencing HCU amongst Aboriginal cardiac patients in central Australia (Figure 1). Our analysis suggests that barriers stem primarily from communication issues arising at all four levels (*Individual, Interpersonal, Primary Care and Hospital System*) and systemic barriers emanating from the *Primary Care* and *Hospital System* levels. Barriers experienced at the systemic levels impacted on the quality of care received by patients, reinforced *Individual* level barriers and created dependency on alternate support at the *Interpersonal* level. Although the study aimed to explore both facilitators and barriers to HCU, participants’ accounts indicate that Aboriginal cardiac patients face significant barriers to HCU. Quotations corresponding to the appropriate participant group are identified to support interpretive validity [29].

### Individual level influencers

#### Language

English was often a second, third or even forth language for many patients, whose first language was typically one of the local Aboriginal languages. Participants explained the need for information to be relayed in their own language to ensure accurate understanding of their illness and the medical concepts and terms used*.*

Staff’s inability to speak local languages enhanced patient’s feelings of fear, as they were being spoken to in a language they did not understand regarding important issues, such as their illness. Poor communication led them to misinterpret, or not fully comprehend, what was being explained or what they should expect.

*“She gets frightened cause she doesn’t know what the doctor is talking about, you know. She might have to have an operation and she don’t know.”* [Non-cardiac community member]

#### Knowledge of illness

Cardiac patients seemed to know little about their illness or signs and symptoms associated with having a cardiac event. Patients desired information but the limited nature of clinician-patient communication meant patients often stayed uninformed.

*“I thought that they might find out what was wrong with me in [hospital], but they just said, ‘You’ll be fine, you’ll live a long time.’ That’s not much, you know. I want to know what’s wrong with me, inside my body.”* [Aboriginal cardiac patient]

Most cardiac patients had no idea they were having a cardiac event. Their knowledge of the signs and symptoms of a cardiac event revolved around “typical” symptoms, such as chest pains. Having a limited understanding of their illness affected patients’ perceived need for HCU at the time of their event and following discharge.

#### Perceived need

Many patients believed they didn’t need to seek health services following their cardiac procedures. This was influenced by a lack of cardiac education and suboptimal communication between the health systems, clinicians and patients, and clinicians and patients’ family. Consequently, a prominent factor discussed amongst patients was the idea of being “fixed” or cured following a cardiac procedure.

*“They opened for my blockage, oh, what, maybe a half an hour, fifteen minutes? They took photo of me first, you know, that blockage, and then when they opened them, those blockages, they took those pictures after. And then they showed me before and after. Before, it was all blocked and after, it was all clear.”* [Aboriginal cardiac patient]

One patient referred to his angioplasty procedure as “magic” and disclosed that he had not gone to a follow-up appointment since his cardiac event, 7 months prior. Limited understanding of one’s illness and cardiac procedures affected patients’ decisions to seek follow-up care or take medication(s) to manage their chronic condition.

#### Past experiences

Aboriginal people’s oral culture was a powerful means of transmitting previous health system experiences to family and community members. Having had a significant other who accessed health services and subsequently died invoked a strong deterrent for HCU amongst surviving partners and relations. Many participants inferred a causal association between HCU and death, particularly when patients were transferred to interstate hospitals.

*“Well, as soon as they hear the word [hospital] they expect…the worst. Cause a lot of family we’ve had down that way, have passed away. So they don’t really want to hear about [hospital].”* [Non-cardiac community member]

#### Mistrust

Negative past experiences, language barriers, perceived racism, and a lack of cultural awareness contributed to Aboriginal participants’ mistrust in the health system. Patients did not fully comprehend their treatments or the western biomedical model and therefore ascribed the high levels of mortality amongst Aboriginal people to clinicians’ mistreatment.

*“Cause a lot of Aborigine people passing away, you know, and they think that doctors are doing something to them.”* [Aboriginal cardiac patient]

However, patients seemed to trust health care providers who had been around for a long time, as they had a better understanding of Aboriginal issues and knew how to interact with patients.

*“You can tell the nurses that been in Alice Springs forever and those nurses that are new, you can pick it out just by how them talking to you. And mob people come to pick that nurse, keeping ask for that nurse.”* [Aboriginal liaison officer]

#### Fear

Virtually all Aboriginal participants shared stories of fear in regards to HCU. Fears were fostered by multiple factors such as, clinicians’ poor communication skills and lack of cultural competence, impacting on participants’ uncertainty about the nature of their illness and treatment options, and negative past experiences. For some, the fear was so pervasive that they completely refused to utilise health services.

*“… family friend, grew up with us, he had to go down to [hospital]. It was life or death for him, [but] he kept putting it off, he was very frightened. He was a very educated, highly educated bloke. By the time he ended up going down there [hospital], he ended up dying on the operating table.”* [Aboriginal health worker]

#### Competing priorities

HCU was also influenced by competing priorities, which included social issues, family obligations associated with Aboriginal culture and obligations to attend grieving ceremonies. Participants explained how individuals often prioritised family and community affairs ahead of their own individual health. Many women worried about leaving their children and/or grandchildren to go to the hospital. This was reflected in the converging perspectives of health care providers and non-cardiac community members.

*“We try again to explain, you know, ‘you gotta think about yourself sometimes …you’re really sick, you gotta go [to the hospital].’ And a lot of response I get is ‘who’s gonna look after them [children] when I go?”* [Aboriginal health worker]

#### Avoidance relationships

Avoidance relationships guide social and personal interactions within the Aboriginal kinship system [30]. Barriers to seeking care surfaced when patients were in a direct avoidance relationship with the attending Aboriginal clinician or liaison officer. Participants spoke of waiting for another staff member before using health services.

*“Yah, I can’t talk if he’s my relation. He can’t tell me, but if he’s somebody else, then yes, he can tell me all the story of how he’s feeling and I’ll explain it to the doctor there. If he’s my cousin or something, he can’t tell me nothing.”* [Aboriginal liaison worker]

#### Basic needs

Few cardiac patients or non-cardiac community members living in town camps or remote area communities possessed a telephone or vehicle. Non-cardiac community participants recounted stories of Aboriginal people who died prior to reaching the hospital because they lacked these basic needs.

*“… then she died, that old woman, died right there … I brought her where she want to go [hospital], but nothing, she died in the car, that old woman I picked up along the way.”* [Non-cardiac community member]

### Interpersonal level influencers

#### Communication between clinicians and patients’ family

Family members were usually not involved in patients’ treatment because of the limited communication that arose between clinicians and the patient’s family. Participants stated that family members were typically not present during consultation or contacted when a patient was admitted, transferred to or discharged from a hospital.

*“When someone is going to Adelaide, I [nurse] don’t consciously think, ‘Oh, does all his family know that he’s going to Adelaide?’ That is something we don’t, I don’t do. It’s something I actually haven’t even thought of.”* [Health care provider]

As a result, many family members were unaware of the patient’s cardiac illness, the required medical procedures or follow-up care. Such communication issues are a concern given the benefits family involvement can offer, as emphasised by one participant:

*“The benefits go back to the family because then the family understand what this illness is, the patient understands, and if the husband can’t understand English, the wife will interpret… and so, everybody can get that one picture of what the doctor talking about.”* [Non-cardiac community member]

#### Alternate support

Due to the presence of communication issues and systemic barriers existing at the health system level, patients often relied on family and community members, kin or other patients for alternate support. Individuals providing alternate support, helped patients negotiate health services, acted as interpreters by explaining medical procedures and kept patients company during hospital transfers and admission. Alternate support played a fundamental role in encouraging patients to use health services.

*“A lot of people don’t want to come [to hospital] unless they got family members.”* [Aboriginal health worker]

Further, cardiac patients who had previously utilised health care services and who, therefore, had some knowledge of how to negotiate the health care system, also acted as a form of alternate support.

*“When I got down there [hospital], I slept at residence wing because I knew where to go now. There were some other people from out bush and they didn’t know where to go, so I said ‘Just follow me, I’ve been here before.’”* [Aboriginal cardiac patient]

### Primary care and hospital system level influencers

Given the similarity of influencers operating at the *Primary Care* and *Hospital System* levels, they are presented in a combined section, below; when an influencer operates at only one level, that exception is noted.

#### Communication between clinicians and patients

Miscommunication frequently occurred between clinicians and patients. Clinicians often explained the nature of participants’ illness, cardiac procedures, and medications using complicated medical jargon. Participants explained that they desired information be communicated to them clearly and expressed frustration at not being able to comprehend the information relayed to them.

*“Well, sometimes I can’t understand them, they talking that type of language they learnt in medical school, you can’t, you don’t know that. I tried to look up a few words in the dictionary; it’s not there, it’s just not there.”* [Aboriginal cardiac patient]

Health care providers also acknowledged this gap in communication, highlighting the additional challenges involved when a common language was not spoken.

*“I don’t think the majority of the time when I explain things to people they understand at all what I’m telling them.”* [Health care provider]

One health care provider explained that communication skills were not commonly taught in medical school; instead, it was something that developed with experience.

*“Actually the best thing is ‘now you tell me, repeat back what you understood, and ’have you got any questions?’ and…‘do you want me to talk to family?’ and that only comes, I think, with exposure and learning. I think that’s something as medical professionals, as doctors, we don’t get taught.”* [Health care provider]

Absent or inadequate explanations also affected patients’ understanding and adherence to prescribed medications. This meant that patients were often sent home without a clear understanding of how or when to take their medications.

*“I take my tablets, don’t know what tablets they give me, the nurse just got tablets for me from pharmacy and sent me home. They supposed to be giving you tablets and you know, telling you what to do when you get back home and all that, but nothing.”* [Aboriginal cardiac patient]

#### Communication between the primary care and hospital systems

In many instances communication *between* the health systems was compromised, impacting on patients continuity of care. Patients’ medical records were not routinely transferred to primary care services or hospitals and health care providers described acquiring patients’ medical records as “rare” and “a big bonus”.

*“The cardiology team down in Adelaide often say ‘follow up by the cardiologist… [in Alice Springs]’, and it never quite gets communicated for whatever reason, and so suddenly you find six months later that this patient has not been seen by anybody.”* [Health care provider]

As further expressed by Aboriginal cardiac patients and family members it was not uncommon for patients to receive cardiac care in Adelaide, return to their hometown in Alice Springs and fail to receive follow-up care.

#### Organisation

##### Wait times and inflexible hours

Long waiting times and inflexible hours often deterred patients’ decisions to use health services.

*“What you find is that Aboriginal people get up and walk out. They’ll wait maybe five or six hours and then say, ‘Ah stuff this, I’m going.’”* [Non-cardiac community member]

##### Intake procedures (exclusive to Hospital System level)

Participants often experienced problems scheduling appointments and with necessary accommodation; in some instances, the receiving hospitals were unaware that patients were being transferred to them.

##### Continuity of care (exclusive to Hospital System level)

Poor management and communication issues *between* the health systems were identified as contributing factors to poor continuity of care. A lack of, or inadequate, discharge summaries meant that clinicians were unaware of patients’ diagnoses and previous treatment(s), inevitably affecting follow-up care. Furthermore, poor organisation contributed to patients’ difficulties in obtaining their test results. Patients were uncertain of their health status which delayed necessary treatment(s) and lead to participants considering HCU a “waste of time”.

*“There was no appointment made for me to go back to hospital. I don’t even know what my results was. I went to [primary care service] and told one of them doctors over there and he was following it up for me… this was a few months ago now.”* [Aboriginal cardiac patient]

As explained by a health care provider, patients seemed to fall in a “black hole” following their cardiac procedure with few receiving specialised follow-up care. These negative experiences reinforced mistrust in the health system and consequently deterred HCU.

#### Availability and delivery of health services (exclusive to Hospital System level)

##### Escort eligibility

“Escorts” are family members or kin who accompany patients when transferring to, or leaving, a hospital. Their involvement improves patients’ agreement to being transferred to tertiary hospitals. Escort policies were limited primarily to underage patients or those with special needs. Restrictive escort policies and resource constraints often impeded escort eligibility, impacting patients’ decisions to seek tertiary care.

*“That’s why, you know that stuff when family members can go with them [to hospital], it’s really important, otherwise you wouldn’t get half of what you get now, and you probably don’t even get most people now! You know, with that family member, more likely that they’ll go.”* [Aboriginal liaison officer]

One health care provider explained that limited escort eligibility was largely due to minimal resources available to patients, proving it even more challenging to accommodate family members. High demands and limited resources meant patients often had to fly alone.

*“There is a limited resource and we haven’t got unlimited number of planes. And I guess that’s where a lot of the pressure comes and why a lot of people [escorts] get refused.”* [Health care provider]

The escort systems’ guidelines were not adapted to meet Aboriginal people’s needs. Consequently, particular issues, such as language barriers and patients’ fears associated with travelling alone were neglected.

*“These are special circumstances and I think they’ve been forgotten”* [Health care provider]

##### Cardiac specialists/services

Limited availability of cardiac services in remote communities meant many patients did not receive appropriate care. The lack of a cardiac unit and minimal cardiac specialists in remote areas and in town acted as a significant barrier, impeding HCU. Absence of cardiac services affected cardiac patients’ continuity of care; some participants described cases where patients refused treatment in town so they could return to their family in remote area communities.

*“They have to come to town to get treatment. I’ve seen people give up, go back to community, and die.”* [Aboriginal health worker]

##### *Cardiac education*

Cardiac patients stated that they were often afraid of and/or anxious about undergoing necessary cardiac procedures because procedures were not properly explained. Cardiac education was limited to watching a “very westernised” angiogram video. A cardiac patient described their experience during an angiogram procedure:

*“They shaved my groin but they never done the test through the groin. They done the test, they put the tube through here (pointing to wrist and then elbow) and I didn’t know what to do. I was full naked on a big operating bed, just with a blanket over me and a plastic over my arm, and I was thinking, if they want to do something with my arm, then why am I full naked?”* [Aboriginal cardiac patient]

An important part of the post discharge management of cardiac disease is enrolment in a cardiac rehabilitation program where emphasis is on secondary prevention and health education. However, cardiac patients described receiving minimal or no cardiac education following their discharge. Health care practitioners also described the limited cardiac rehabilitation available:

*“I think at the moment there is 15 hours of cardiac education a week, for the whole of Alice Springs. So that’s 200 events a year, 15 hours.”* [Health care provider]

Minimal information was available for cardiac patients to understand their illness, their medication and required lifestyle changes to prevent subsequent cardiac events and other related co-morbidities. This reinforced individual level barriers such as patients’ knowledge of their illness and negatively influenced perceived need for HCU.

#### Transportation services

Most participants did not own a vehicle. Although a bus service was provided by primary care services in town, it was highly inadequate and unreliable. At the *Hospital System* level, patients arriving at Alice Springs airport from Adelaide stated that no hospital transportation service was available to them; patients were often left stranded and unable to return to their homes.

#### Cultural awareness

Non-Aboriginal health care providers received minimal or no cultural awareness training compromising the quality of care provided, their ability to effectively communicate with Aboriginal patients and adapt services to meet Aboriginal patients’ needs.

*“Some of the resident doctors out bush tried to develop a better orientation with cultural mentorship. They have someone in the community responsible for teaching them about cultural aspects which is so much more appropriate [but it takes] more than one day. Well, if you are not here for more than a few weeks, off you go, you’re done and you’ve had no [cultural] training.”* [Health care provider]

Issues with cultural appropriateness were exemplified by the health system’s inability to provide gender-appropriate care, which is highly regarded amongst Aboriginal people, inevitably deterring HCU when not available.

*“If a male wants a male, well, then a male doctor gotta see that person. You know, that’s what I see all the time. When that old lady talking language, I hear her, I just tell that [male] doctor, ‘She don’t want to see you, she want to see a female doctor’”.* [Aboriginal liaison officer]

Cultural oversights impacted upon patients’ negative past experiences and mistrust of the system, reinforcing individual level barriers.

#### Aboriginal liaison officers

Aboriginal liaison officers (ALO) - government-funded interpreters and cultural workers- represent one solution to clinician-patient language barriers and help reduce patients’ fears and anxieties.

*“The liaison team explained what’s gonna happen and said he’s [ALO] gonna be there for them, so then they jumped in the car and went.”* [Aboriginal liaison officer]

Although ALOs were described to facilitate HCU, they were rarely available.

*“Especially that emergency department too, hey? There is not one interpreter there… and [patients] can’t understand, they’re just nodding their head.”* [Non-cardiac community member]

Converging storylines arising from the narratives suggested that greater resource support was needed to increase ALOs availability. Health care practitioners also described the absence of standard guidelines to determine if, or when, a patient required an ALO.

*“You can’t have an interpreter there all the time. It’s only for important things, like when you know, you are going to chop someone’s foot off.”* [Health care provider]

#### Racism

Racism was also perceived as a factor influencing HCU. Almost all Aboriginal participants described feeling as though they were not offered the same health services as non-Aboriginal patients.

*“It’s ‘You do it this way’, ‘This is the best treatment for you’ and a white person will come in with the same thing and it’s ‘You got this choice and you got this choice, what do you want to do?’”*[Aboriginal health worker]

This was also described by a health care professional:

*We certainly use [interpreter services] well enough if it’s a European that comes [to hospital]. A German speaking person…we automatically go get that [interpreter services] straight away or find someone to do it. But if we want a Pitjantjatjara, or an Alyawarr speaking person, you know, we can use the interpreter speaking services, but we don’t.* [Health care provider]

Further, ambulance drivers’ resistance to pick up patients living in town camps or remote area communities depicted a strong example of institutionalised racism.

*“There is a lot of stigma from the health service providers about entering into town camps, especially at night time.”* [Non-cardiac community member]

Aboriginal participants felt that their treatment was affected by widely held stereotypes about Aboriginal individuals:

*“People think that ALL Aboriginal people drink [alcohol], that’s why we get treated the same way, as the drunks in town.”* [Non-cardiac community member]

Discrimination could be sensed as reinforcing individuals’ mistrust and negative past experiences with the health system.

#### The Western biomedical model

The health care system’s emphasis on the western biomedical approach to health care was perceived to negatively influence HCU by Aboriginal people. The illness focus of this model was viewed as not able to account for social, psychological, cultural or behavioural dimensions of health relevant to Aboriginal people. It was seen as conflicting with Aboriginal people’s holistic notion of health which emphasises relationships between people, places and things. Patients and community members expressed concerns over the perceived lack of attention of clinicians to building relationships with patients.

*“You need to develop a relationship, make it about people, gain trust.”* [Non-cardiac community member]

#### Health system support

Some patients and community members reported that the health system did not acknowledge the living situations and cultural aspects of Aboriginal people’s lives, and thus, could not adequately support their needs. The system was described as “top-down,” with Aboriginal people uninvolved in health service development and implementation.

*“Giving people the power to be able to say, ‘Well, we want this’ and then resource those ideas. Putting the money behind what the people themselves are saying, not listening to white fella, who don’t know nothing about Aboriginal people.”* [Non-cardiac community member]

Aboriginal participants stressed the importance of having a sense of ownership and involvement in the development of health services targeted towards them, to make them effective and functional within the community.

### Quality of care

Communication issues arising on all four levels and health system level barriers presented at the *Primary Care* and *Hospital System* levels contributed to sub-optimal quality of care for cardiac participants.

Lack of enabling factors (or inhibiting factors) predominately influenced HCU at the Primary Care and Hospital System levels, while predisposing factors primarily influenced Individual level factors. The presence of an enabling factor, Alternate Support, at the Interpersonal level linked the health system and the individual, thereby facilitating use of health services. HCU is a function of all these factors, acting either separately, or in combination. Negative experiences or barriers to HCU impacted on the quality of care received by Aboriginal cardiac patients, affected individuals’ subsequent decisions to use health care services and health outcomes.

## Discussion

This study identified a variety of factors perceived to influence Aboriginal patients’ HCU from the time of their cardiac event to 6–12 months post-event. The main findings and contributions of this work can be summarised in five key points.

The first contribution is the development of a culturally sensitive HCU matrix that is adapted to the needs of Aboriginal people living in central Australia. The matrix addresses important factors influencing HCU not previously identified for socially disadvantaged populations, such as Aboriginal people. For instance, Schepper’s (2006) adaptation of Andersen’s model [19] does not address racism as a barrier to HCU among ethnic minorities, even though it is noted that such populations are often victims of discrimination and segregation. Institutional racism in the Australian health care system has previously been documented [31,32] and our findings demonstrate that patients or stories of significant others who experienced racism are less likely to use health services in the future. Similarly, issues such as previous experiences, mistrust, fear and competing priorities are not included in the above-mentioned HCU frameworks. In the present analysis, these factors represented important barriers among Aboriginal people and need to be considered in efforts aimed at improving HCU. Participants described communication issues at various levels as the most important factor influencing HCU. Andersen (1995) does not address communication problems as a potential barrier to HCU and although Scheppers (2006) lists communication issues at the patient and provider level, the role family members play in facilitating clinician-patient communication and the health system’s need to provide appropriate services to ensure and support intercultural communication is not dealt with. Andersen’s model uses a Westernised approach to understanding HCU and does not consider barriers such as lack of cultural awareness or need for family involvement. To our knowledge there is no existing research that specifically addresses the barriers to HCU among Aboriginal cardiac patients in Australia.

Second, even though this study aimed to assess barriers and facilitators to HCU, the emphasis of participants’ stories primarily reflected barriers to HCU even where facilitators such as ALOs were noted. In this instance, where ALO’s were present, participants emphasised the insufficient number of ALOs. Most barriers encountered at the health system level thus point to *limited* or *absent* enabling factors (or inhibitors), such as *lack* of cultural awareness amongst health care providers and *minimal* ALOs [33]. Focus should be placed on providing system level supports to enable improved HCU and thus effect change at the *Individual* level. This interpretation is consistent with health promotion strategies that emphasize changing environments to enable improved interactions between individuals and the systems with which they interact [20].

Third, this study indicates that Aboriginal cardiac patients have a poor knowledge of their illness which affects the management of their chronic illness, perceived need for future HCU, and overall health outcomes. Low levels of health literacy have been found to negatively affect health outcomes and is considered to be a more accurate predictor of health status than determinants of health such as, socioeconomic status, education, employment, race or gender [34]. Low literacy skills also predict the degree with which individuals engage in the health system and their understanding of their chronic illness [35]. There is an urgent need for health literacy to be integrated at the system level to improve patients’ quality of care and health outcomes. Upstream approaches of integration include developing policies and practices to support health literacy initiatives that are culturally appropriate and sensitive, ensuring organisational development and ongoing capacity building opportunities for health staff.

Fourth, the findings reveal a severe breakdown in cardiac patients’ continuity of care following discharge. Various communication and system level barriers affect patients’ ability to receive test results, follow-up care and cardiac rehabilitation services. A disjunction between *Primary Care* and *Hospital System* services means that most cardiac patients fail to receive essential post-event health services. The Australian National Health Performance Committee identified continuous care as one of the nine domains required for effective quality health performance [36]. Management of chronic illness such as CVD requires continuous clinical care, which incorporates a patient centred approach that is adapted to patients’ needs and supports self-management. Future research and action in this area is warranted.

Fifth, our findings point to the importance of family involvement as patients’ alternate support systems, assisting where the health care system unequivocally fails. The health care system’s expectation for family members to accurately interpret complex medical terminology without training is unrealistic and leads to misinterpretation and communication breakdown, in some cases extreme and ultimately life-threatening [37-39]. Family involvement should be offered to patients systematically during consultations, hospital admissions and transfers, in informed consent procedures and following discharge where important lifestyle modifications, regular follow-up care and adherence to medication are essential to support self-management and positive patient outcomes.

The factors influencing HCU identified in this study were confirmed in a recent pilot study focusing on improving Aboriginal cardiac patients’ journey for those living in remote areas of the Northern Territory [15]. The potential for positive improvements in HCU were demonstrated through small and inexpensive systemic changes to the health system, e.g. employment of a full-time remote liaison nurse and the development of culturally appropriate clinical pre- and post- surgery procedures. The factors influencing health care utilisation and impacting on quality of care for Aboriginal patients have been addressed in a recent article, which proposes a collaborative model of hospital based care to improve health service delivery for Aboriginal patients [40].

Study limitations include the time constraint of a four-month data gathering period. A longer period may have provided for more information from participants and recruitment of greater numbers of Aboriginal cardiac patients. This study did not recruit family members or kin of Aboriginal cardiac patients who died *prior* to accessing health services, out of respect to the deceased and their families. It is possible, however, that Aboriginal cardiac patients who died prior to accessing health services may have encountered the greatest barriers to HCU. Study results might therefore under represent the extent of the barriers faced by Aboriginal cardiac patients. The use of a fundamental qualitative descriptive design to guide the study relied on individual’s own perceptions to explain a situation, while directly influencing their own use of health services. As such, these perceptions may not fully explain the factors affecting HCU. The development of a culturally sensitive matrix for HCU is limited by being derived from a single study. The matrix provided a sound method of categorising participants’ stories; however it is not an exhaustive list of all possible factors influencing HCU among Aboriginal cardiac patients in central Australia. Future research in this area is warranted to further build on the findings, to ensure a comprehensive and complete culturally sensitive framework that maps the relationships between the factors influencing HCU at various levels and its impact on health outcomes. Transferability of results requires caution as findings may not be representative of Aboriginal people and cardiac patients, and health care providers in other settings in rural and remote Australia.

## Conclusion

Aboriginal people living in and around Alice Springs face significant barriers to HCU at multiple levels and domains of experience. Communication issues and system level barriers negatively reinforce *Individual* level barriers and thus impact Aboriginal people’s abilities and/or decisions for HCU. Due to these barriers, Aboriginal cardiac patients receive sub-optimal quality of care, significantly risking subsequent cardiovascular events. Efforts to improve HCU amongst Aboriginal cardiac patients must more effectively consider their experiences, abilities, circumstances and culture.

## Competing interests

The authors declare that they have no competing interests.

## Authors’ contributions

All authors have made significant contributions to the published study. SA, MD, and AB were involved in the conception and design of the research study. SA and AB were involved in data collection. SA transcribed and analyzed all primary documents. AB, MD, and MC aided in the interpretation of the results. SA, MC and MD drafted and revised the manuscript. All authors have read and approved the final manuscript.

## Pre-publication history

The pre-publication history for this paper can be accessed here:

http://www.biomedcentral.com/1472-6963/13/83/prepub
